# Editorial for the Special Issue “Microplastics in Aquatic Environments: Occurrence, Distribution and Effects”

**DOI:** 10.3390/toxics10070407

**Published:** 2022-07-21

**Authors:** Costanza Scopetani, Tania Martellini, Diana Campos

**Affiliations:** 1 Faculty of Biological and Environmental Sciences Ecosystems and Environment Research Programme, University of Helsinki, Niemenkatu 73, FI-15140 Lahti, Finland; 2 Department of Chemistry “Ugo Schiff”, University of Florence, Via della Lastruccia 3—Sesto Fiorentino, 50019 Florence, Italy; tania.martellini@unifi.it; 3 Consorzio Interuniversitario per lo Sviluppo dei Sistemi a Grande Interfase (CSGI), University of Florence, Via della Lastruccia 3—Sesto Fiorentino, 50019 Florence, Italy; 4 CESAM—Centre for Environmental and Marine Studies, Department of Biology, University of Aveiro, 3810-193 Aveiro, Portugal; diana.campos@ua.pt

The large production and widespread daily consumption of plastic materials—which began in the last century—together with the often-inadequate collection and recycling systems, have made plastics and, consequently, microplastics (MPs) ubiquitous pollutants [1].

The scientific community is increasingly concerned about microplastic pollution and its possible effects on biota and the environment. Aquatic ecosystems such as rivers, lakes, estuaries, seas, and oceans seem to act as important sinks for plastics and microplastics. Microplastic pollution is so widespread that we might assume no aquatic environment has been left untouched [2,3,4,5].

Microplastic pollution as a global concern is confirmed by the research papers collected in this Special Issue; these papers come from 28 Universities and research institutions and are spread across ten countries in three continents.

The Special Issue “Microplastics in Aquatic Environments: Occurrence, Distribution and Effects” collected and published 11 novel contributions focusing on microplastics in aquatic environments, their occurrence and distribution, and the effects they might have on the environment and biota. The selected papers comprise three reviews and eight research articles. In their review, Yang et al. (2021) [6] summarized the current literature on MPs in the marine environment, focusing on the sources and fates of MPs and their impacts on marine organisms; moreover, they highlighted the potential of bacteria in plastic degradation processes and the need to further study this subject.

Santini et al. (2022) [7] addressed the occurrence of natural and synthetic microfibers in waters, sediments, and biota in the Mediterranean Sea, emphasizing the challenges in distinguishing natural fibers from plastics ones, and the need to further study the environmental impact of both.

Lim et al. (2022) [8] conducted a meta-analysis of the characterization of plastic ingested by fish on a global scale, and found that plastic fibers are the most-ingested items (70.6%). Additionally, the authors observed that polyethylene (15.7%) and polyester (11.6%) are the most abundant polymers found in fishes’ digestive organs. In terms of size, the most frequently ingested plastics were small microplastics (<1 mm).

The eight selected research papers can be grouped into three main themes: (1) the effects of microplastic exposure to aquatic biota (rotifers, mussels, fish larvae, and microalgae), encompassing 55% of the published papers in this SI [9,10,11,12,13,14]; (2) the distribution and seasonal variation of microplastics in aquatic environments [15]; and (3) the contaminants associated with microplastics in freshwater environments [16].

As Guest Editors of this Special Issue, we were pleased to receive several papers concerning the interaction between microplastics and biota; despite a large number of peer-reviewed papers published on this research topic, there are still several gaps that need to be filled [17,18]. Zhang et al. (2022) [12], for instance, investigated the toxicity of fluorescent nano- and microplastics (80 nm and 8 μm) on grass carp embryos and larvae using scanning electron microscopy (SEM) and fluorescence imaging. Their results showed that nanoplastics accumulated in the chorion and did not penetrate the embryo’s chorionic membrane. The larvae were prone not only to ingesting microplastics and expelling them with their feces, but also to ingesting the expelled microplastics again while feeding on their own excrement, re-accumulating the plastic particles in their oral cavities. Furthermore, the authors showed that microplastics around 1 μm in size could accumulate in the larvae’s nasal cavities.

Drago and Weithoff (2021) [9] analyzed the fitness responses of two rotifer species, *Brachionus calyciflorus* and *Brachionus fernandoi*, when exposed to polystyrene (1-, 3-, 6-µm), polyamide microplastics (5–25 µm) and silica beads (3 µm, SiO_2_). The results showed that 3-µm polystyrene had a significant effect on the population growth rate of both rotifer species, whereas no effect was evidenced after exposure to polyamide microplastics and silica beads.

In another study, von Hellfeld and co-authors (2022) evaluated the toxicity of polystyrene MPs in marine mussels *Mytilus galloprovincialis* when exposed to two different polystyrene microplastic sizes (45 µm and 4.5 µm) [10]. The exposure was carried out with pristine and contaminated microplastics, with cadmium (Cd) and benzo(a)pyrene (BaP). The pristine microplastics (both tested sizes) were found in the digestive gland after 1 day of exposure, while after 3 days of depuration, 4.5 µm microplastics had accumulated within the gill filaments. In contrast to Cd, BaP body burdens increased significantly in mussels exposed to BaP-contaminated microplastics, causing histological changes in the digestive gland. These results show that polystyrene microplastics can act as a carrier of organic contaminants and pose a threat to aquatic biota.

The toxicity of microplastics on *M. galloprovincialis* was studied also by Rodrigues et al. (2022) [11]. Mussels were exposed to polyamide microplastics alone and in combination with the toxic exudate from the invasive red seaweed *Asparagopsis armata*. The study showed that microplastics accumulated mainly in the digestive gland of the organisms and that the combined exposure to microplastics and *A. armata* induced oxidative damage at the protein level in the gills and reduced the production of byssus. This study highlights the need to assess microplastics’ toxicity in combination with other stress factors, such as invasive species and contaminants. In this regard, Scott et al. (2021) [16] studied the interactions between different polymer types of microplastics and per- and polyfluoroalkyl substances (PFAS) in a lacustrine and a controlled environment. The polymers were kept submerged in the lake water in the presence of associated organic/inorganic matter and biofilm for one and three months; meanwhile, in the laboratory experiment, the polymers were kept in water contaminated with PFAS but without inorganic and organic matter. The results indicated that the presence of inorganic and organic matter considerably enhances the adsorption of PFAS by polymers; this emphasizes the need to assess the risks posed by microplastic pollution under realistic environmental conditions.

All the exposure experiments described so far suggest that microplastic pollution may constitute a serious hazard to aquatic biota. For instance, according to Hadiyanto et al. (2021) [13], Styrofoam microplastics can inhibit the photosynthesis process of *Spirulina platensis*, as well as being a source of nutrients, especially carbon, for the microalgae.

Other organisms that have been found to be capable of ingesting microplastics are blackfly larvae (Simuliidae), as shown by Corami et al. (2022) [14]. Two species of blackfly larvae, *Simulium equinum* and *Simulium ornatum*, were sampled from two rivers in Italy and analyzed for microplastics (<100 μm), and natural and non-plastic synthetic fibers. The authors showed, for the first time, that blackfly larvae can ingest microplastics from their habitat and suggested that these organisms could be employed as bioindicators for microplastic pollution in rivers, as they are already bioindicators used to assess river water quality. Indeed, rivers can be heavily contaminated with microplastics, as Wicaksono et al. (2021) pointed out in their study [15]. The authors collected water and sediment samples along the Tallo River (Indonesia) during the wet and dry seasons. Microplastic concentration was up to 3.41 ± 0.13 item/m^3^ and 150 ± 36.06 item/kg for water and sediment samples, respectively. As in many other aquatic environments, the most abundant polymers found in the Tallo River were polyethylene and polypropylene [15].

The results of the contributions collected herein have helped to fill some knowledge gaps about the occurrence, distribution, and effects of microplastics on aquatic ecosystems. The outcomes clearly indicate that microplastic pollution is a serious environmental issue; the scientific community should increase its knowledge and understanding of how it could affect the environment, biota, and humans, and how it could be reduced and prevented. Nevertheless, to adopt adequate mitigation strategies and contribute to preserving biodiversity and environmental health towards zero pollution, it is pivotal that the studies consider realistic and environmentally relevant conditions.

We would like to thank all the authors for submitting their original contributions to this Special Issue. We greatly appreciate the support of all the reviewers who spent time evaluating and improving the quality of the manuscripts. Last but not least, we would like to thank the editors of *Toxics* for their kind invitation, and Mia Yan, Selena Li, and Linda Li of the *Toxics* Editorial Office for their precious support.
